# The effect of expectation on satisfaction in total knee replacements: a systematic review

**DOI:** 10.1186/s40064-016-1804-6

**Published:** 2016-02-24

**Authors:** Timothy Barlow, Tamsyn Clark, Mark Dunbar, Andrew Metcalfe, Damian Griffin

**Affiliations:** Clinical Sciences Research Laboratories, University of Warwick, University Hospitals of Coventry and Warwickshire, Clifford Bridge Road, Coventry, CV2 2DX UK

**Keywords:** Satisfaction, Expectation, Knee replacement

## Abstract

Total knee replacement has reliably been shown to have a beneficial effect in knee osteoarthritis; however, around 17 % of patients are dissatisfied with the result. A commonly proposed mechanism driving the dissatisfaction rate is a discrepancy between expected and actual/perceived outcome. Our aim was to conduct a systematic review examining any association between pre-operative expectations and satisfaction. A comprehensive electronic search strategy was used to identify studies from MEDLINE, EMBASE, and the Cochrane Library from inception until May 2015. Data was extracted according to PRISMA guidelines and an online, published protocol. Four studies are included in this review. One study found an association between expectations and satisfaction. Different measures of expectation and satisfaction were used in all studies. To date, there is no consensus on how expectations or satisfaction should be measured, and a large number of studies that have the available information failed to conduct the relevant sub-group analysis. Further elucidation and consensus of how to measure expectations and satisfaction around joint replacement would aid this area of study greatly. On the basis of the current evidence it appears expectations have a small effect, if any, on satisfaction after knee replacement.

**Electronic supplementary material:**

The online version of this article (doi:10.1186/s40064-016-1804-6) contains supplementary material, which is available to authorized users.

## Background

Primary osteoarthritis (OA) of the knee is a condition that can lead to loss of knee function, pain, and deterioration in quality of life. This in turn can lead to difficulty working, performing activities of daily living, stress, and depression (Smith and Zautra 2008). Ten percent of the U.K. population over the age of 55 suffers from pain as a result of knee osteoarthritis (Peat et al. 2006). With an ageing population this condition will present more and more of a health burden.

Total knee replacement has reliably been shown to have a beneficial effect (Juni et al. 2006), and over 90,000 knee replacements were performed in England and Wales in 2014, with over 90 % of these for OA (Registry 2014). However a sub group of patients exist that have poorer outcomes following knee replacement. Some studies show dissatisfaction rates as high as 17 % (Hawker et al. 2009).

The question of what factors drive this high rate of dissatisfaction is one that has received much attention in the literature. Most previous work has focused on the effect of different prostheses and surgical factors on outcome and this has only been able to account for a small proportion of the variability in outcome (Callahan et al. 1994; Judge et al. 2012). There is growing evidence that factors intrinsic to the patient may significantly affect outcome (Santaguida et al. 2008). Such factors include psychological factors, demographics, and patient expectations (Heck et al. 1998; Lingard et al. 2004; Mannion et al. 2009).

Investigations into expectations are complex, as different authors describe different constructs under the term expectation (Haanstra et al. 2012). For the purposes of this paper, we describe expectations as “cognitions regarding probable future events” (Haanstra et al. 2012). This specifically excludes self efficacy, which can be defined as “beliefs in one’s capabilities to organize and execute the courses of action required to produce given attainments” (Bandura 1977). A further clarification needs to be made between expectations and expectation fulfilment. Pre-operatively a patient has expectations (a series of beliefs about the outcome of the operation), post-operatively a patient can decide if those expectations have been met or not (expectation fulfilment) (Scott et al. 2012). Using these definitions, it is logical that patients whose expectations are not fulfilled are likely to be less satisfied. This has been demonstrated in the literature (Scott et al. 2012), but a further layer of complexity exists when we consider the shift in patients’ view of what is healthy or to be expected over the course of their treatment (response shift) (Razmjou et al. 2006). Even with this response shift, expectation fulfilment is linked to outcome after knee replacements (Scott et al. 2012; Clement et al. 2014).

However, what remains unclear is the relationship between pre-operative expectations and satisfaction. Indeed, the potential for altering satisfaction based on appropriate management of pre-operative expectations may be a worthwhile approach to tackling the issue of high dissatisfaction rates. It will also provide clinicians with information on how critical managing expectations are when satisfaction is the endpoint of interest. While previous attempts at synthesising the evidence exist (Haanstra et al. 2012; Dyck et al. 2014; Waljee et al. 2014), none have set out to answer this basic and fundamental question. Specifically, most reviews have not had satisfaction as the outcome of interest, and included both knee and hip replacements together (Haanstra et al. 2012; Dyck et al. 2014; Waljee et al. 2014). These operations differ not only by outcome (and, one would expect, by pre-operative expectation) (Registry 2014), but also by patient demographics and potentially aetiology (Grotle et al. 2008; Jokela et al. 2013). Therefore, when examining expectations, combining these operations is potentially flawed.

The aim of this systematic review is to determine if pre-operative expectations affect post-operative satisfaction in knee replacements.

## Patients and methods

In line with Preferred Reporting Items for Systematic Reviews and Meta-Analyses (PRISMA) guidelines (PRISMA 2012) the protocol was submitted to the PROSPERO database prior to the performance of the systematic review. The reference number is CRD42015023216 and is available at http://www.crd.york.ac.uk/PROSPERO/display_record.asp?ID=CRD42015023216.

### Search strategy

A comprehensive electronic search strategy was used to identify studies from MEDLINE (Medical Literature Analysis and Retrieval System Online, Bethesda, Maryland, USA), EMBASE (Excerpta Medical Database, Amsterdam, The Netherlands) and the Cochrane Library using all available data from their inception until May 2015.

The search strategy is available in the supplementary materials and was designed to be as comprehensive as possible in order to mitigate the risk of producing ‘precise but spurious results’ (Egger et al. 1998) (Additional file 1).

### Eligibility criteria

Studies were eligible for inclusion if they were prospective studies observing the effect of expectation on satisfaction in knee replacement for osteoarthritis. Studies where information was retrieved from a database were also included if the pre-operative expectation was collected prospectively. Case series and case–control studies were not included as there is a significant potential for selection bias with these types of study design (Sara et al. 2009). Only studies that were presented in English were included in the analysis.

The subjects included in each study were patients about to undergo total knee replacement with a diagnosis of osteoarthritis. Where patients with other arthritic diseases (e.g. traumatic arthritis, inflammatory arthritis), or patients who had undergone revision knee replacement, made up more than 5 % of the study population the studies were excluded. However, studies containing mixed groups of patients were included if there was subgroup analysis that clearly differentiated the population of interest. Studies that included both hip and knee replacement patients were only included where there was a subgroup analysis of the knee patients. Authors of papers that had both hip and knee replacement patients, but where no subgroup analysis was presented, were contacted and asked if any subgroup analysis was done, and to forward it on if it had been.

Any study that collected information on patient expectations retrospectively was excluded as this has been shown to be unreliable (Razmjou et al. 2006).

### Expectation and satisfaction measurements

Many tools exist for the measurement of expectation and satisfaction. No paper was excluded on the basis of its measurement method, although these are commented on.

### Analytic methods

Studies had to have made some attempt to associate satisfaction with expectation. Various methods of regression or correlation analysis were acceptable as long as they were clearly explained in the methods section in order to determine that their use was appropriate for the type of data collected.

### Quality assessment

The Newcastle Ottawa Scale provides a system for assessing quality across three domains: selection, comparability, and outcome (Stang 2010). Each domain is scored with a star rating system. A summary score is not provided in an attempt to provide data on the biases inherent in the study design.

### Data extraction

After the initial search was performed the studies were screened for eligibility in sequential rounds where their relevance was assessed using at first their titles and abstracts, and finally full review of the paper. Two reviewers (TC and TB), who were both experienced in performing systematic reviews, then independently re-examined each full paper to ensure that they met the inclusion criteria. Relevant data was extracted and a quality assessment performed independently by both reviewers, resolving any differences through discussion and review.

Due to the nature and heterogeneity of the analyses used in the studies, combined with the heterogeneity of the measurements of expectations and satisfaction, no formal meta-analysis was performed.

## Results

### Description of studies

**Fig. 1:**
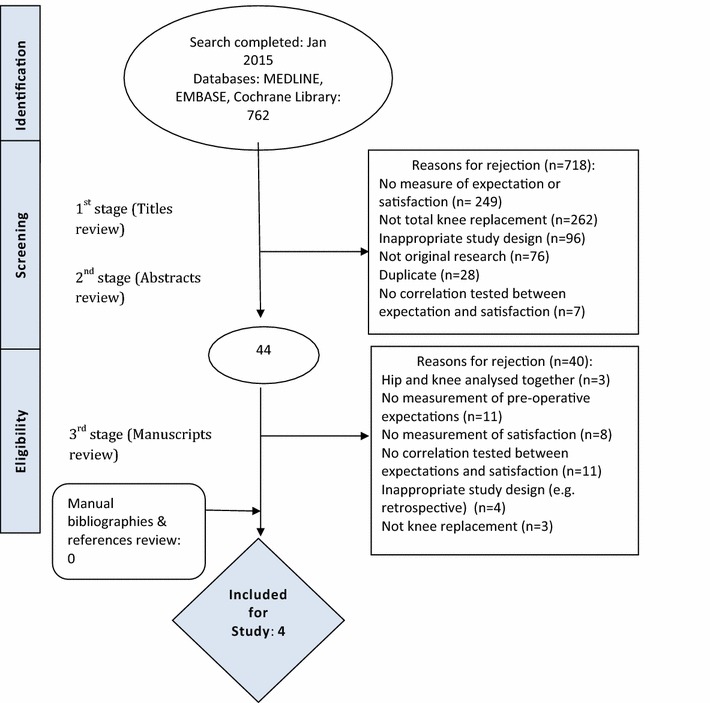
Flow chart of included studies

Our search returned 762 studies. Of these some were duplicates from multiple databases that due to a technical limitation had to be removed by hand during the title search. Figure 1 is a flow chart detailing the studies that were excluded. We have included four studies in this review (Table 1).

**Table 1:** Included studies

Author	Year	Title	Study design	Sample size	Time to follow up	Measure of expectation	Measure of satisfaction	Analysis	Link between expectation and satisfaction
Kiran et al.	2015	Variations in good patient reported outcomes after total knee arthroplasty	Cohort	365	2 years	Two questions on a 3 and 4 part Likert scale	Global satisfaction (yes/no)	Univariate	No
Lingard et al.	2006	Patient expectations regarding total knee arthroplasty: differences among the United States, United Kingdom, and Australia	Cohort	598	1 year	Four questions on a 4 part likert scale	Four questions using a four part likert scale	Multivariate	No
Mannion et al.	2009	The role of patient expectations in predicting outcome after total knee arthroplasty	Cohort	87	2 years	Three questions with free text and likert scale	Global satisfaction four part likert	Univariate and multivariate	Univariate model—yes Multivariate model—no
Vissers et al.	2010	Functional capacity and actual daily activity do not contribute to patient satisfaction after total knee arthroplasty	Cohort	44	6 months	Three questions on a four part likert scale	Global satisfaction five part likert scale	Univariate	No

### Quality assessment

Table 2 describes the quality assessment using the Newcastle Ottawa scale. All studies were judged to be of similar quality.

**Table 2:** Quality assessment

Study	Selection	Comparability	Outcome
Kiran et al. (2015)	4	0	1
Vissers et al. (2010)	4	0	2
Mannion et al. (2009)	4	1	1
Lingard et al. (2006)	4	1	2

### Expectations and satisfaction

Of the four studies included in the review the sample size varied from 44 to 598. A different method of measuring expectations was used in each study, and a different method for measuring satisfaction was used in each study (please see Additional File 2 for description of measures used). One study was international in nature and was also the largest by almost double the sample size (Lingard et al. 2006). All other studies were single centre (Kiran et al. 2015; Vissers et al. 2010; Mannion et al. 2009).

Three studies conducted a univariate analysis (Kiran et al. 2015; Vissers et al. 2010), with only one study finding an association between pain and function expectations and satisfaction (Mannion et al. 2009).

Two studies conducted a multivariate analysis and neither found a significant association between expectations and satisfaction (Lingard et al. 2006; Mannion et al. 2009). One study had the largest sample size and did not conduct univariate analysis (Lingard et al. 2006). The second study, that had found an association in the univariate model, did not find an association in the multivariate model; however, they had a sample size of 87, with over 5 covariates in the model, and had included expectation fulfilment in the multivariate model (Mannion et al. 2009). It is therefore questionable if this study had the necessary power to conduct this analysis, and the logic of including expectation fulfilment (which would be expected to account for the same variation in the final model as pre-operative expectation) is questionable.

Two studies reported positive finding of expectation related to other outcome measures. Kiran found that pre-operative expectation of no pain gave higher (better) OKS score (p < 0.01) (Kiran et al. 2015), and Lingard found that pre-operative expectation of no pain resulted in better WOMAC pain scores (p = 0.039) and pre-operative expectation of not needing a walking aid resulted in better WOMAC function score (p < 0.0001) (Lingard et al. 2006).

## Discussion

Overall, one study out of four found an association between expectation and satisfaction. This could be due to: each study measuring slightly different constructs of “expectation” and “satisfaction”; the effect of expectation on satisfaction may be small, and of dubious clinical relevance.

This study has highlighted several issues surrounding expectation and satisfaction in knee replacements. The first key finding is the multiple measures used by each study to evaluate expectation and satisfaction. There is a great deal of difficulty in measuring these constructs, and multiple theoretical models exist to try and explain some of the complexity. For example the latent state-trait theory suggests that with measurement instruments we measure a persons state, which will depend upon the person (the person’s traits), the situation, and the interaction between the person and situation (Steyer et al. 1999). This highlights the difficulty in measuring these constructs consistently and accurately, and may go some way to explain the low numbers of studies examining this key issue. It seems likely that until the orthopaedic community can reach a consensus on what is important to measure, and how to measure it, progress will be slow.

The second key issue is the low number of studies that have examined this issue within the literature. Many studies have examined expectation fulfilment (Scott et al. 2012; Clement et al. 2014; Kumar et al. 2015; Adie et al. 2012), and there were multiple studies that measured pre-operative expectation and satisfaction but were excluded from this review because they did not make any attempt to test for an association (Suda et al. 2010; Clement et al. 2014; Kumar et al. 2015), or they included hips and knees as one cohort (Gonzalez Saenz de Tejada et al. 2014; Brokelman et al. 2008; Gandhi et al. 2009). This is a key finding is itself, as pre-operative expectations would be an ideal target for modification if an association were present. It may be that the difficulty surrounding reliable measurements of these constructs is responsible.

Limitations of this review include the lack of formal meta-analysis, which was not possible due to the heterogeneity within the papers included. English language articles were specified in the inclusion criteria for full paper review, but not in the search strategy. Therefore, although the possibility of missing important information from other sources existed, in practice there were no fully published papers in other languages that would have met the inclusion criteria.

There was some variation in the length of follow up between studies. This has the potential to introduce a form of timing bias as it has been shown that the functional status of knees after replacement can improve for around 2 years following surgery (Pynsent et al. 2005). Studies that did not show associations in some domains might well have done so if the length of follow up was extended.

Systematic reviews are always subject to publication bias and in particular the delay or lack of publication of studies with negative findings (Stern and Simes 1997). We have tried to minimise this by including all archived, published research from each of our searched databases, but the possibility remains that some evidence may remain unpublished. This is more likely from older, less well-designed studies, as the resources required performing a high quality modern, observational study would dictate publication irrespective of the findings. As the concepts and subsequent measurement of expectations and satisfaction are relatively new, it is likely we have included all relevant studies.

Some other studies have been conducted examining the association between expectation in hip and knee and satisfaction. Of these, a study conducted by Gonzalez et al. that was multicentre and included a multivariate analysis demonstrated a significant effect of expectations on satisfaction (Gonzalez Saenz de Tejada et al. 2014), whereas two others, with smaller sample sizes and from single centres, did not (Brokelman et al. 2008; Gandhi et al. 2009). This does tend to suggest that the construct of expectation and satisfaction that is being measured is key and can alter the significance of the result.

A further issue surrounds that of the clinical significance of our result. Certainly, with regard to satisfaction, managing a patient’s pre-operative expectation appears to have little effect on post-operative satisfaction. However, this is not the whole picture, as outcome can be measured in a multitude of different ways. Patient Reported Outcome Measures (PROMs) show associations with pre-operative expectations in multiple studies (Kiran et al. 2015; Lingard et al. 2006; Mahomed et al. 2002), and expectations are associated with length of stay and discharge destination (Halawi et al. 2015a, b).

## Conclusion

One out of four studies found evidence of an association between pre-operative expectations and satisfaction. While this could be interpreted as either no effect, or a small, clinically insignificant effect, we would caution against a move to abandon management of patient expectations for two reasons: expectations and satisfaction are difficult constructs to measure, and the papers included here may be measuring different aspects of these constructs; satisfaction is not the only outcome measure available, and other outcome measures are related to pre-operative expectations. One of the major findings of this paper is the lack of large, high quality, studies that have examined this key issue, and the lack of consensus within the literature on how to measure expectation and satisfaction.

## Additional files

10.1186/s40064-016-1804-6 Search strategy.
10.1186/s40064-016-1804-6 Measures of expectation and satisfaction.
