# Supplementary material for: The effect of expectation on satisfaction in total knee replacements: a systematic review
Source: Springerplus. 2016 Feb 24;5:167. doi: 10.1186/s40064-016-1804-6 (PMC4766134; doi:10.1186/s40064-016-1804-6)
Supplement: Supplementary file 1 — 10.1186/s40064-016-1804-6 Search strategy. [file 40064_2016_1804_MOESM1_ESM.docx]

**Additional file 1. Search strategy**

Pubmed

| **Set** | **Search terms** |
| --- | --- |
| #1 | "Arthroplasty, Replacement, Knee"[Mesh] OR (("Arthroplasty"[Mesh:NoExp] OR "Arthroplasty, Replacement"[Mesh:NoExp]) AND ("Knee"[Mesh] OR "Knee Joint"[Mesh] OR)) OR "Osteoarthritis, Knee/surgery"[Mesh:NoExp] OR "Osteoarthritis, Knee/therapy"[Mesh:NoExp] OR "Knee Prosthesis"[Mesh] OR ("Knee Arthroplasty"[tiab] OR "knee replacement"[tiab] OR "joint arthroplasty"[tiab] OR "knee osteoarthritis"[tiab] OR "knee prosthesis"[tiab] NOT medline[sb]) |
| #2 | "Health Knowledge, Attitudes, Practice"[Mesh] OR expectation[tiab] OR expectations[tiab] OR expectancy[tiab] OR expectance[tiab] OR credibility[tiab] OR "patient preference"[tiab] OR OR "health knowledge"[tiab] OR "health attitude"[tiab] OR "attitude to health"[tiab] OR belief[tiab] |
| #3 | #1 AND #2 |
| #4 | #3 NOT ("addresses"[Publication Type] OR "biography"[Publication Type] OR "case reports"[Publication Type] OR "comment"[Publication Type] OR "directory"[Publication Type] OR "editorial"[Publication Type] OR "festschrift"[Publication Type] OR "interview"[Publication Type] OR "lectures"[Publication Type] OR "legal cases"[Publication Type] OR "legislation"[Publication Type] OR "letter"[Publication Type] OR "news"[Publication Type] OR "newspaper article"[Publication Type] OR "patient education handout"[Publication Type] OR "popular works"[Publication Type] OR "congresses"[Publication Type] OR "consensus development conference"[Publication Type] OR "consensus development conference, nih"[Publication Type] OR "practice guideline"[Publication Type]) NOT ("animals"[MeSH Terms] NOT "humans"[MeSH Terms]) |

Embase search

1. knee arthroplasty/ or arthroplasty/

2. total knee replacement/

3. knee surgery/

4. knee prosthesis/

5. knee osteoarthritis/

6. 1 or 2 or 3 or 4 or 5

7. expectation/

8. expectancy/

9. attitude to health/

10. patient attitude/ or patient preference/

11. 7 or 8 or 9 or 10

12. 6 and 11

13. limit 12 to (human and english language)

14. limit 13 to (article or book or editorial or journal or report)

Cochrane library search

- #1 Enter terms for search arthroplasty arthroplasty  5948
- #2 Enter terms for searc replacement replacement 18235
- #3 Enter terms for searc knee knee 13026
- #4 Enter terms for searc arthroplasty OR replacement AND knee arthroplasty or replacement and knee 6624
- #5 Enter terms for searc (#1 OR #2) and #3 (#1 or #2) and #3 3837
- #6 Enter terms for searc expectation or expectancy expectation or expectancy 4481
- #7 Enter terms for searc #5 and #6 #5 and #6 53
