# Supplementary material for: The effect of expectation on satisfaction in total knee replacements: a systematic review
Source: Springerplus. 2016 Feb 24;5:167. doi: 10.1186/s40064-016-1804-6 (PMC4766134; doi:10.1186/s40064-016-1804-6)
Supplement: Supplementary file 2 — 10.1186/s40064-016-1804-6 Measures of expectation and satisfaction. [file 40064_2016_1804_MOESM2_ESM.docx]

Additional file 2. Measures of expectation and satisfaction

| Author | Expectation measure | Satisfaction measure |
| --- | --- | --- |
| Kiran | Two questions (usual activities and Pain) on a 3-part Likert scale | Global satisfation (yes/no) |
| Lingard | Four questions (pain level, walking distance, limi- tation of recreational activity, and use of a walking aid) on a 4 part likert scale. | Four questions using a 4 part likert scale. |
| Mannion | Three questions (expected time until full recovery (open answer, in months), expected pain after recovery from surgery (not at all painful through to very painful), and expected limitations in everyday activities after recovery from surgery (not limited at all through to greatly limited). | Global satisfaction 4 part likert |
| Vissers | Three questions: a) pain after surgery b) limitations of activities of daily liv- ing after surgery and c) the overall success of the operation) on a four part likert scale | Global satisfaction 5 part likert scale |
